# Unfolding the Endoplasmic Reticulum of a Social Amoeba: *Dictyostelium discoideum* as a New Model for the Study of Endoplasmic Reticulum Stress

**DOI:** 10.3390/cells7060056

**Published:** 2018-06-10

**Authors:** Eunice Domínguez-Martín, Mariana Hernández-Elvira, Olivier Vincent, Roberto Coria, Ricardo Escalante

**Affiliations:** 1 Instituto de Investigaciones Biomédicas “Alberto Sols” (CSIC-UAM), Arturo Duperier 4, 28029 Madrid, Spain; edominguez@iib.uam.es (E.D.-M.); ovincent@iib.uam.es (O.V.); 2 Departamento de Genética Molecular, Instituto de Fisiología Celular, Universidad Nacional Autónoma de México, 04510 Ciudad de México, México; melvira@email.ifc.unam.mx (M.H.-E)

**Keywords:** *Dictyostelium*, endoplasmic reticulum, endoplasmic reticulum stress, unfolded protein response, inositol-requiring enzyme 1 (IRE1)

## Abstract

The endoplasmic reticulum (ER) is a membranous network with an intricate dynamic architecture necessary for various essential cellular processes. Nearly one third of the proteins trafficking through the secretory pathway are folded and matured in the ER. Additionally, it acts as calcium storage, and it is a main source for lipid biosynthesis. The ER is highly connected with other organelles through regions of membrane apposition that allow organelle remodeling, as well as lipid and calcium traffic. Cells are under constant changes due to metabolic requirements and environmental conditions that challenge the ER network’s maintenance. The unfolded protein response (UPR) is a signaling pathway that restores homeostasis of this intracellular compartment upon ER stress conditions by reducing the load of proteins, and by increasing the processes of protein folding and degradation. Significant progress on the study of the mechanisms that restore ER homeostasis was achieved using model organisms such as yeast, *Arabidopsis*, and mammalian cells. In this review, we address the current knowledge on ER architecture and ER stress response in *Dictyostelium discoideum*. This social amoeba alternates between unicellular and multicellular phases and is recognized as a valuable biomedical model organism and an alternative to yeast, particularly for the presence of traits conserved in animal cells that were lost in fungi.

## 1. *Dictyostelium* as a Model Organism for Experimental Biology Research

Owing to its simplicity and easy genetic manipulability, some microbial organisms prove to be powerful biology-research tools; for instance, *Saccharomyces cerevisiae* is one of the most widely studied eukaryotic organisms. Much of the current knowledge in biochemistry, and molecular and cellular biology arose from research performed with this yeast. However, since this fungal organism has some specific genetic, cellular, and metabolic traits that are not widely conserved, other eukaryotic microbial organisms emerged to address cellular processes that diverged greatly in yeast cells. One of these organisms is *Dictyostelium discoideum*, a social soil-dwelling protist, taxonomically classified in the Amoebozoa phylum, the sister group to animals and fungi [1]. Despite its phylogenetic classification, *Dictyostelium* displays cellular processes that are conserved in animal cells, but that are absent in fungal or plant cells, such as phagocytosis and chemotaxis. Interestingly, it also has traits that are conserved in fungi and plants, but were lost in animal cells, such as phosphorelay signaling systems, and cellulose production [2,3].

**Figure 1:**
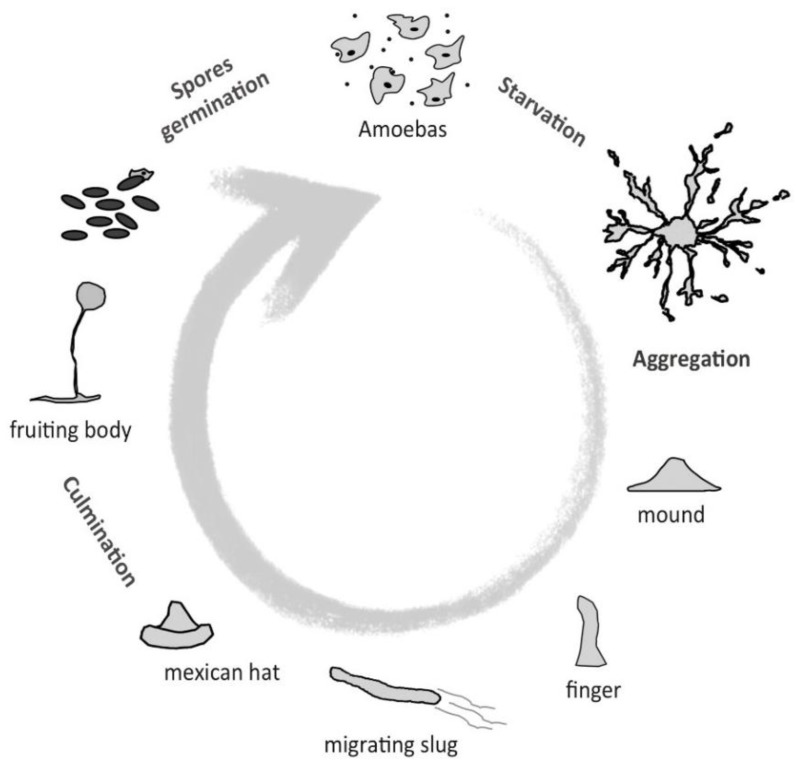
Diagram of the *Dictyostelium* life cycle. Individual amoebas feed on yeast and bacteria, and multiply via fission. When nutrients are scarce, cells aggregate and undergo a developmental program, comprised of distinct stages that culminate in the formation of a fruiting body, which is composed of a stalk, and a sorogen filled with spores. Under suitable environmental conditions, the spores germinate.

*Dictyostelium* has a life cycle that alternates between unicellular and multicellular phases, depending on nutrient availability (Figure 1). As unicellular amoebas, they obtain nutrients from phagocytizing yeast or bacteria, and they multiply via fission about every 8 h. Remarkably, under starvation, *Dictyostelium* cells stop mitotic division, and start an intercellular signaling communication process mediated by the secretion of various molecules. One of them, the cyclic adenosine monophosphate (cAMP), acts as a chemoattractant that triggers the polarization, migration, and aggregation of groups of about 10^5^ cells from the species *Dictyostelium discoideum*. These aggregates of apparently homogeneous cells enter a developmental program that generates a multicellular organism with distinct cell types. After various developmental stages, including one as a multicellular motile slug, the *Dictyostelium* differentiation program culminates in the formation of a fruiting body composed of a sorogen filled with spores, which is supported by a cellulose stalk made of dead cells (a detailed review of this process was addressed in Reference [4]).

Since its first isolation and description more than 80 years ago, a growing number of studies used *Dictyostelium discoideum* to unravel diverse biological questions [2,3]. The genome of this haploid organism is fully sequenced [5], and many different techniques were developed allowing the study of a wide range of topics including infection and drug testing [6]. In addition, *Dictyostelium* was used in basic studies of cell and developmental signaling, among them, the pathways involved in endoplasmic reticulum (ER) homeostasis, maintenance, and regulation [7]. Conditions that interfere with ER homeostasis contribute to the pathogenesis of human chronic disorders including diabetes and some neurodegenerative syndromes, such as Alzheimer’s, Parkinson’s, and Huntington’s diseases [8,9]. *Dictyostelium* emerged as an advantageous model for the study of signaling pathways involved in neurodegeneration (reviews on this topic were addressed in References [10,11,12]). In addition, this amoeba is an interesting model for the study of pathways involved in neurological disorders associated with protein aggregation, since it efficiently regulates the accumulation of prion-like protein aggregates [13,14].

## 2. The Endoplasmic Reticulum of a Social Amoeba

The ER is the largest eukaryotic organelle. This complex membranous network is the place where essential functions such as protein folding and modification, lipid synthesis, and calcium (Ca^2+^) storage are fulfilled. In the following sections, a general comparative description of the current knowledge on the *Dictyostelium* ER structure and function is presented. Table 1 contains a summary of all the *Dictyostelium* ER proteins that were discussed throughout this text.

### 2.1. A Membranous Network with an Intricate Structure

Two domains that maintain luminal continuity can be identified in the ER, the nuclear envelope (NE) and the peripheral ER, each with a particular structure and specific characteristics. In the NE, two stacked membranes of low curvature form the inner and outer nuclear membrane (INM and ONM), whereas the peripheral ER spreads across the cytosol, shaped by a network of interconnected tubules and flat sheet-like regions [15,16]. In mammalian cells, an array of constricted tubule clusters, enriched with three-way junctions, forms the peripheral ER matrices. This set of structures is relatively flat, and has a heterogeneous composition and topology [16]. In addition, there are some specialized ER regions formed through flattened membranes, which can be found shaping the nuclear envelope, in the perinuclear region or close to the plasma membrane [17,18,19]. A tubular network that expands from the NE and these matrices forms a system that connects all the ER domains. The tubules’ surface is highly curved, which facilitates surface-dependent functions such as lipid synthesis, and signaling between the ER and other organelles [15].

**Figure 2:**
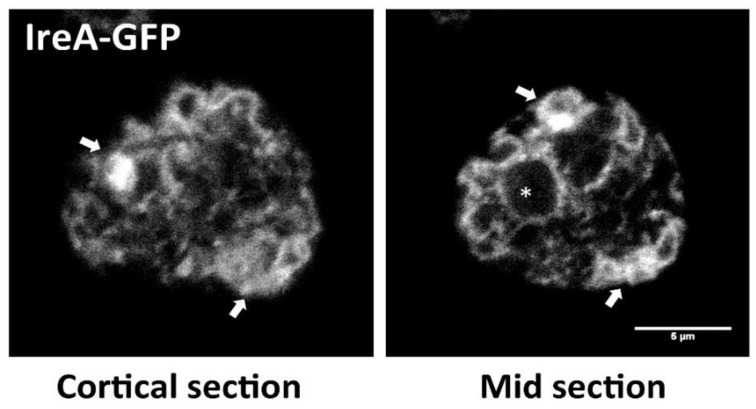
The *Dictyostelium* endoplasmic reticulum (ER). In vivo confocal microscopy pictures showing a cortical section and a mid-section of a wild-type (WT) cell expressing the ER marker, Inositol requiring enzyme A (IreA) fused to the GFP. The asterisk pinpoints the nucleus, surrounded by the perinuclear ER. Arrows highlight zones where sheet-like regions are evident. Tubules can be distinguished across the entire cell area. (Scale bar represents 5 μm).

The *Dictyostelium* ER can be visualized by expressing inositol-requiring enzyme A (IreA) tagged with a fluorescent protein. IreA is an ER-resident protein that participates in the unfolded protein response (UPR) pathway (see Section 3.1—The IreA Branch). The *Dictyostelium* ER can be divided into an NE region and a peripheral zone, consisting of a network of tubules and sheet-like regions, which spread throughout the cell (Figure 2). Domains with sheet-like appearance are distributed mainly in peripheral cell zones, and in close proximity to the NE, while tubules are spread throughout the cell, and can be observed as more defined structures in medial cell sections. 

In mammalian cells, the curvature of the tubules is maintained by a highly conserved integral membrane family of proteins, referred to as reticulons (RTNs) [20]. RTN proteins contain a reticulon homology domain (RHD) at their C-terminus which is formed by two long hairpin transmembrane domains, separated by a hydrophilic linker [21]. These hairpins are inserted in the cytoplasmic leaflet of the ER membrane to provoke membrane bending. RTNs can also oligomerize in order to determine the diameter of the tubules [20,21,22].

Studies on how the *Dictyostelium* ER structure is maintained are still scarce; however, a homolog of the reticulon family was phylogenetically identified (annotated as *rtnlc*/DDB_G0293088) [23]. Owing to the presence in *Dictyostelium* of single orthologs of some of the proteins involved in the maintenance of ER architecture, it represents an advantageous model for combined genetic studies on ER dynamics and structure. For instance, reticulons were implicated in neurodegenerative disorders [24], but the study of this protein family in mammalian cells is challenging since they contain a large number of isoforms. 

### 2.2. The ER Is a Dynamic Structure Continuously Rearranged

The ER is a highly dynamic network; its architecture is modified according to specific cellular demands or processes, such as changes in cellular morphology, cell migration, mitosis, and upon stressful conditions. For instance, specialized secretory cells require an increase in the number of sheet-like structures to synthesize large amounts of proteins, while adrenal, liver, and muscle cells require an ER network predominantly formed by tubules [25].

The synthesis of new tubules in the ER, together with the maintenance of its network and dynamics, depends greatly on the association of the ER with the cytoskeleton [26,27,28], while the maintenance of the reticulated network requires continuous events of contact and fusion between tubules. ER fusion events are mediated by atlastin (ATL) proteins, a dynamin-related family of GTPases that mediate homotypic membrane fusion [29,30]. During the fusion events, two GTP-bound ATLs, localized at opposing membranes, transdimerize, and GTP hydrolysis induces a conformational change that pulls the ER membrane close enough to fuse [31,32]. Plants and yeasts possess functional homolog GTPases of ATLs [33,34]. 

Recently, the role of the *Dictyostelium* ATL homolog Synthetic enhancement of *YOP1* (Sey1) was evaluated during infection with the intracellular bacteria *Legionella pneumophila* (*L. pneumophila*) [35]. This pathogen exploits a conserved replication mechanism in mammalian macrophages and in *Dictyostelium* cells, based on the construction of a special ER-derived compartment known as a *Legionella*-containing vacuole (LCV). Interestingly, in *Dictyostelium* cells, Sey1 modulates *L. pneumophila* replication, possibly by mediating homotypic membrane fusion at later steps of LCV maturation.

### 2.3. A Well-Connected Membranous System

The ER interacts dynamically with other membranes, such as the plasma membrane, and has contacts with other organelles such as mitochondria, the Golgi body, and endosomes, among others. These interactions, known as membrane contact sites (MCSs), allow the transport of ER-synthesized lipids and Ca^2+^ to other organelles. In addition, MCSs are involved in organelle biogenesis, distribution, inheritance, and maintenance (a more extensive review on ER contact sites can be found in Reference [36]).

In yeasts, the mitochondria–ER membrane contacts (MERCs) are tethered by the ER–mitochondria encounter structure (ERMES), which organizes the contact between the ER and the mitochondrial outer membrane. Similarly, the mitochondrial contact site and cristae organizing system (MICOS) enables the contact between the mitochondrial inner and outer membranes. Together, both systems are referred to as the ER–mitochondria organizing network (ERMIONE). In addition, there is an heteromeric hexamer known as the ER membrane protein complex that participates in diverse ER processes, and in the tethering of MERCs, where it is presumably involved in phosphatidylserine traffic (a review with an evolutionarily analysis on the topic was given in Reference [37]). 

In yeasts, the ERMES system comprises an ER transmembrane protein, maintenance of mitochondrial morphology-1 (Mmm1p), and a cytosolic protein, mitochondrial distribution and morphology 12 protein (Mdm12p), which form a complex with two outer mitochondrial membrane proteins, Mdm34p and Mdm10p [38]. Strikingly, orthologs of this complex are absent in mammalian cells, but proteins such as mitofusins, phosphofurin acid cluster sorting protein 2 (PACS2), the mitochondrial voltage-dependent anion channel (VDAC), and the vacuole membrane protein 1 (VMP1), among others, were implicated in the regulation of this contact site; however, a conclusive outlook on how MERCs are tethered in these organisms remains elusive [39,40].

In *Dictyostelium*, the architecture of the ER contact sites with other membranes has not been described. However, the ER–mitochondria contact sites may be regulated by a homolog of the fungal ERMES, since an ortholog of the Mdm12p ERMES protein was recently identified in this amoeba, and was purified for structural analysis [41]. In addition, ortholog genes of *mmm1* (DDB_G0285921), *mdm10* (DDB_G0278805), and *mdm34* (DDB_G0274475) were predicted and are annotated in the *Dictyostelium* genome database [42].

Interestingly, the *Dictyostelium* genome encodes a VMP1 ortholog. This ER transmembrane resident protein is conserved in plants and animals, but it is absent in yeast. *Dictyostelium vmp1* knock-out mutant cells show severe ER and Golgi structural alterations, together with a set of pleiotropic phenotypes, ranging from autophagy defects to deficient osmoregulation [43,44]. The phenotypes of *vmp1^−^* cells may be a consequence of the severe imbalance in ER homeostasis. Studies using this amoeba were crucial in unraveling the role of this still poorly understood ER protein and paved the way for further studies on other ER proteins that are conserved in *Dictyostelium*, but not in yeast cells.

### 2.4. The Main Source of Lipid Synthesis

The ER and the Golgi body are the major sites of membrane lipid synthesis in eukaryotic cells. Lipid synthesis occurs in specialized ER regions rich in tubules, and in vesicles that are adjacent to the Golgi body, called the ER–Golgi intermediate compartment (ERGIC) [45]. Once lipids reach the ERGIC, they are transported to their final destination through contact with other organelles, or via vesicle transport [46].

*Dictyostelium* total lipids are partitioned into approximately 60% phospholipids and 40% neutral lipids [47]. The membranes of this amoeba display a composition similar to that observed in mammalian cells, where phosphatidylserine, choline, and ethanolamine are the major constituents [47]; therefore, it can represent a comparative model for the study of ER lipid-associated processes. Of note, *Dictyostelium* contains several unsaturated fatty acid species, with stigmastenol as the major steroid [47,48], and ether inositol phospholipids [49]. As in other organisms, *Dictyostelium* maintains lipid homeostasis by packing excess lipids as inert neutral species sheltered in vesicles that emerge from the ER, which are referred to as lipid droplets (LDs). Upon addition of palmitic acid and cholesterol, *Dictyostelium* cells accumulate LDs which contain triacylglycerol (TAG) and steryl esters in an approximate ratio of 1:15, similar to the ratio observed in mammalian adipocytes [50]. These vesicles contain about 72 proteins, including perilipin, which is implicated in protecting LDs from lipolysis. Perilipin which is conserved in mammals, but absent in yeast and *Caenorhabditis* [51], has a homologous gene in *Dictyostelium*, *plnA.*

Analogously to mammalian organisms, *Dictyostelium* cells contain two acyl coenzyme A (CoA) diacyl-glycerol acyltransferases (DGATs) that participate in the synthesis of TAG. One of these enzymes, Dgat1, localizes to the ER membrane, and provides most of the TAG synthesis activity. As with human DGAT1, this enzyme participates in the synthesis of other lipids such as waxes and ether lipids. The other DGAT enzyme coded for in the *Dictyostelium* genome localizes at lipid droplets (LDs), and has a minor role in total cellular TAG synthesis [52].

### 2.5. A Perfect Compartment to Fold and Modify Proteins

The ER lumen contains a unique glycosylation machinery, and a particular environment enriched with Ca^2+^, with a high oxidizing potential and a high viscosity. All these conditions make up a perfect situation for the synthesis, folding, and modification of integral and secreted proteins.

In order to achieve their functional state, proteins undergo various processes in the ER lumen; these include N-linked glycosylation, disulfide bond formation, folding cycles, and oligomerization.

N-linked glycosylation is a co-translational modification that consists of the addition of an oligosaccharide tree to the asparagine (Asn) residues contained in the Asn-X-serine/threonine motif of proteins. This reaction is triggered by the oligosaccharyl transferase (OST) enzyme, and it is essential for the recruitment of carbohydrate-binding factors in the ER lumen that stimulate protein folding. It serves to increase protein stability by masking hydrophobic stretches or proteolytic cleavage sites, and avoiding back-translocation [53]. Although the *Dictyostelium* OST enzyme is unstudied, it was inferred through comparative studies that it is formed by at least seven subunits, and that it shares similarity with its plant and fly homologs [54].

ER luminal chaperones help the newly synthesized proteins to reach their active conformation by promoting their folding, and by preventing aggregation. There are two groups of ER chaperones: the heat shock proteins (HSP), a protein family that can also be found in all cellular compartments, and the carbohydrate-binding chaperones (CBC), which are specifically located in the ER [55,56].

The ER is mainly enriched with members of the HSP70 and HSP90 families. These proteins recognize specific domains in glycosylated proteins, or exposed hydrophobic segments in the non-glycosylated ones, thus protecting intermediate folded precursors from aggregation by shielding these regions from intermolecular hydrophobic interactions. There is an ER resident HSP70 chaperone, called GRP78/binding immunoglobulin protein (BiP) in metazoans, or Kar2p in yeast, which participates in a broad number of processes besides protein folding [57]. Additionally, the calnexin and calreticulin proteins are two of the main CBCs. These chaperones interact with the glycan moiety to achieve protein maturation and quality control [55].

Orthologs of the broad spectrum of ER-resident folding proteins were identified and described in *Dictyostelium*, among them, the calcium-regulated chaperones—calreticulin and calnexin [58], the HSP70 chaperone—78 kDa glucose-regulated protein (Grp78) [7], and Dd-grp94, a member of the HSP90 family [59].

During the various stages of its life cycle, the *Dictyostelium* ER adapts to continuously changing metabolic demands. As vegetative amoebas, they obtain nutrients primarily via phagocytosis. Through this process, the ER transiently contacts the phagosome during the uptake process. This contact requires calreticulin and calnexin, which participate in Ca^2+^ storage [58]. These observations suggest that the ER participates during phagosome formation, possibly as a membrane source and as a regulator of Ca^2+^ homeostasis.

Two other non-chaperone protein families participate in the ER protein-folding process: the peptidyl-prolyl isomerases (PPIs), which catalyze cis/trans isomerization of peptide bonds, and the protein disulfide isomerases (PDIs) [56,60], a protein family that participates in the formation of disulfide bonds between inter- and intra-chain cysteine residues, a covalent linkage which provides stability to proteins. The formation and disruption of disulfide bonds can also act as a regulatory mechanism for protein activity control. PDI oxidoreductases exchange disulfide bonds with their substrates, which results either in the reduction of its active site and the oxidation of two adjacent cysteines in the substrate to form a disulfide bond, or in the opposite reaction. In yeasts, PDI is kept oxidized through an electron flow pathway catalyzed by the ER oxidase protein 1 (Ero1) [61]. 

The *Dictyostelium* genome codes for two members of the PDI family (*pdi1* and *pdi2*). Remarkably, PDI (*pdi1*) lacks the canonical C-terminus retrieval signal motif for ER localization composed by the amino acids HDEL, and instead, requires a sequence in its last 57 C-terminal amino acids [62]. This motif allows the retention of PDIs at the ER when overexpressed in yeast cells, indicating that this ER-retention mechanism might be conserved in evolution [63]. 

## 3. Endoplasmic Reticulum Stress and the Unfolded Protein Response in a Social Amoeba

To support ER protein homeostasis, the cell must achieve equilibrium between the protein load and the concentration of the ER folding machinery. Conditions that alter this equilibrium may cause defects in protein folding and modification, thus leading to the accumulation of misfolded proteins in the ER lumen, a condition termed as ER stress (ERS) [64]. 

*Dictyostelium* development is regulated via a variety of secreted extracellular signals; it was observed that about 2.6% of the 12,257 proteins predicted to be encoded in its genome are secreted during this process [65]. In addition to the fluctuating metabolic demands of its life cycle, *Dictyostelium* cells encounter various stressful conditions derived from the complex ecosystem it inhabits, some of which cause ER homeostasis imbalances. For instance, this amoeba might share a habitat with the *Streptomyces* species, which evolved competitive survival mechanisms based on the production of antibiotics.

Recently, it was demonstrated that tunicamycin (TN), a fatty acyl nucleoside antibiotic produced by *Streptomyces lysosuperificus* and *Streptomyces chartreusis,* which interferes with N-glycosylation, induces ERS in *Dictyostelium* [7]. In *Dictyostelium*, this antibiotic effectively inhibits protein glycosylation [66]. The TN effects on *Dictyostelium* development were first determined in the early 1980s. Since then, TN was widely used to evaluate the role of N-glycosylated cell-adhesion proteins during the aggregation process. This antibiotic was found to inhibit cellular growth, and to impair development [66,67,68,69,70,71,72,73]. In addition, TN blocks cell fusion during *Dictyostelium* sexual development, mainly due to its effects on glycoprotein production [74]. Interestingly, TN can partially suppress the developmental phenotypes, but not allorecognition impairment, as presented by null-mutants of Transmembrane IPT/IG/E-set repeat protein (TgrB1) and TgrC1, two immunoglobulin-like proteins required for kin discrimination and cell type differentiation during *Dictyostelium* development [72].

After TN treatment, *Dictyostelium* cells undergo evident morphological changes by adopting a spherical shape [7]. Likewise, upon hyperosmotic stress, *Dictyostelium* cells suffer morphological changes due to a cytoskeletal reorganization [75]. Interestingly, a similar rounded morphology was also described for a subpopulation of stress- and detergent-resistant *Dictyostelium* cells, in which the expression of some lipid-metabolism genes were modified [76].

**Figure 3:**
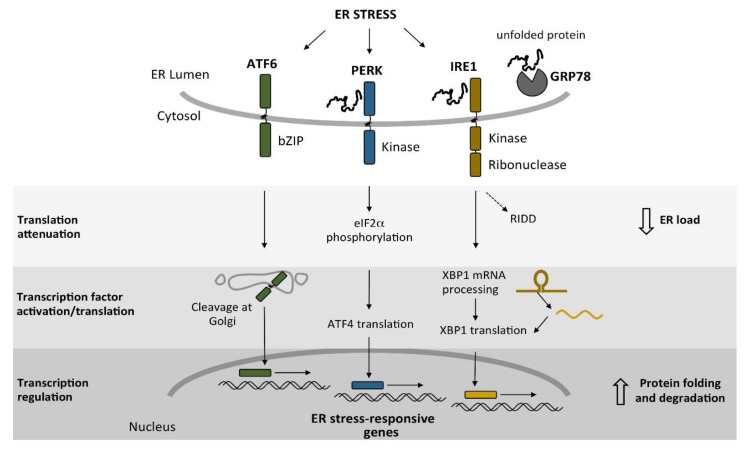
Signaling pathways involved in the unfolded protein response (UPR). In mammalian cells, three signaling branches that depend on the ER transmembrane sensor proteins—activating transcription factor 6 (ATF6), protein kinase RNA-like ER kinase (PERK), and inositol-requiring enzyme 1 (IRE1)—are activated upon ER stress (ERS). PERK and IRE1 can sense ERS by interacting directly with unfolded proteins through their luminal sensor domain. In addition, ATF6, PERK, and IRE1 detect an increase in unfolded proteins when they lose their association with the ER chaperone GRP78/binding immunoglobulin protein (BiP). When these transducers detect ERS, a recovery response is activated. This response mainly regulates two events: the reduction of ER protein load, and an increase in the protein-folding and degradation capacity of the cell. The former is accomplished via translation inhibition, triggered by the PERK-mediated phosphorylation of the eukaryotic initiation factor 2α (eIF2α), and by the degradation of certain messenger RNAs (mRNAs) in the regulated IRE1-dependent decay (RIDD). The second event regulates the activation or translation of transcription factors that, when transported to the nucleus, reprogram transcription to increase the expression of ER homeostatic genes, thus promoting protein folding and modification of the ER.

To cope with ERS, eukaryotic cells evolved a conserved system referred to as the unfolded protein response (UPR), which restores ER homeostasis through the activation of a complex transcriptional program that changes the expression of genes encoding proteins associated with the synthesis of membranes, and for chaperones and degradative enzymes [77]. In addition, the UPR decreases the protein load at the ER through the selective degradation of messenger RNAs (mRNAs) that are targeted to this compartment, and by decreasing the translation rates [78,79]. In metazoans, the UPR is activated in parallel by three ER transmembrane sensors: the inositol-requiring enzyme 1 (IRE1), the protein kinase RNA-like ER kinase (PERK), and the activating transcription factor 6 (ATF6) (Figure 3). Each of these branches senses the folding environment in the ER lumen and activates several transcription factors upon ERS.

After ERS, *Dictyostelium* cells trigger an adaptive transcriptional response, which diminishes the ER protein load, increases ER protein folding, and favours the cellular degradation processes [7]. Interestingly, ERS induces transcriptional changes in some genes that code for proteins of the actin cytoskeleton, and some genes involved in lipid metabolism [7], which suggests that the round cellular morphology caused by ERS might be the consequence of membrane and cytoskeletal rearrangements. Adopting a round morphology devoid of pseudopodia might be advantageous to *Dictyostelium* cells, by aiding in the lipid-balance maintenance in the plasma membrane, and by decreasing energy requirements.

### 3.1. The IreA Branch

As in other organisms, the transcriptional response to ERS in *Dictyostelium* is mediated by the IRE1 pathway, which is, until now, the only UPR signaling branch identified in this amoeba. In plants, yeasts, and mammals, this pathway is constituted by the type I ER-resident transmembrane protein, IRE1, and a basic zipper leucine transcription factor known as the X-box binding protein 1 (XBP1) in mammalian cells, HAC1 in yeast, and bZIP60 in plants.

IRE1, the most conserved UPR transducer, contains an amino N-terminal ER luminal stress-sensor domain, and a carboxy C-terminal cytoplasmic region that harbors a kinase and a kinase extension nuclease (KEN) domain [80]. IRE1 plays a prominent role in the UPR of plants and animals, and it is the only sensor in *Saccharomyces cerevisiae* [81]. Mammalian cells contain two IRE1 isoforms, IRE1α, which is ubiquitously expressed, and IRE1β, which is exclusively expressed in the intestinal and lung epithelia [82,83]. *Arabidopsis thaliana* also has two IRE1 orthologs (IRE1A and IRE1B) that display differential expression patterns [84]. 

When the cell is under ERS conditions, IRE1 is activated via a conformational change triggered by direct interaction with unfolded proteins through its sensor domain and/or by the release of the chaperone GRP78/Kar2p from the same domain. This leads to the formation of high-order IRE1 oligomers, and their transautophosphorylation [85,86,87]. Once activated, IRE1 processes the mRNA of a transcription factor through an unconventional splicing event that eliminates an intron. The processed mRNA is translated into a bZIP that upregulates the transcription of genes encoding ER-resident chaperones and modification enzymes, among others [88]. Although the protein sequence of this transcription factor is poorly conserved between species, the IRE1 cleavage site and the stem-loop structure of the unconventional intron are widely preserved [89,90,91].

In mammalian cells, IRE1 is able to regulate its protein levels via the degradation of its own mRNA. The IRE1 N-terminus can bind to the 5′ end of its own mRNA while the nascent protein is translated and attached to the ribosome. After the release of the nascent protein, IRE1 can dimerize, and activates its RNAse domain to degrade the mRNA [92]. The yeast Ire1p kinase domain participates in the downregulation of the pathway through an autophosphorylation process that requires a 28-amino-acid region in its kinase domain; this hyperphosphorylation destabilizes Ire1p oligomers [93]. 

**Figure 4:**
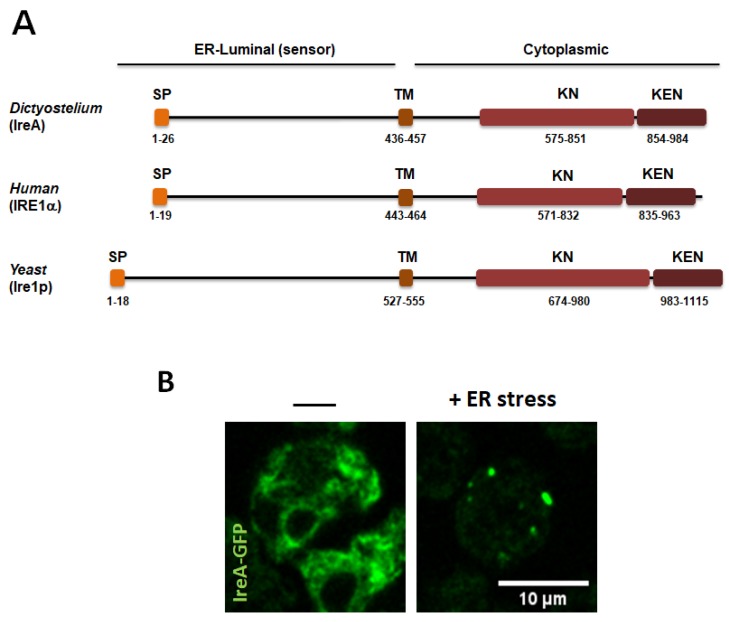
(**A**) Diagram of the structural domains of *Dictyostelium* IreA, compared with its *Saccharomyces cerevisiae* and human orthologs. SP (signal peptide), TM (transmembrane domain), KN (kinase domain), and KEN (kinase extension nuclease domain). Proteins were drawn to scale. Numbers indicate amino acid coordinates. Protein domains were obtained from www.uniprot.org. (**B**) Live-cell confocal microscopy of *ireA^−^* cells expressing the IreA-GFP construct after 4 h, in the absence or in the presence of an ER-stress inducer. The IreA-GFP signal forms large puncta (possibly high-order oligomers). (Scale bar corresponds to 10 μm).

The *Dictyostelium* genome encodes a single IRE1 sensor ortholog, IreA, an ER-localized protein with a single predicted transmembrane domain [7]. IreA comprises two regions, one localized in the ER lumen, which is poorly conserved and likely functions as the ER environment sensor, and the other region that is predicted to be at the cytosolic face of the ER and contains a serine/threonine kinase and a KEN domain (Figure 4A). Both kinase and ribonuclease domains are required in *Dictyostelium* cells to survive ER stress, and both must be fully active to regulate IreA oligomer-formation dynamics during sustained ERS [7]. Upon ER stress, IreA forms transient high-order oligomers (Figure 4B). Oligomerization in the yeast Ire1p is required for the recruitment of unprocessed *HAC1* mRNA [94], and to trigger Ire1p RNAse activation [95]. However, in *Dictyostelium*, the possible transcription factor is yet unidentified.

In animal cells, IRE1 regulates transcript abundance in a branched fashion, since it splices the XBP1 transcript, but also degrades mRNAs through its RNAse domain via a mechanism named regulated IRE1-dependent decay (RIDD) [78,79,96]. The RIDD pathway decreases the ER protein load of secreted and transmembrane proteins, and can activate apoptosis by degrading anti-apoptotic pre-mRNAs [97]. Interestingly, the yeasts *Schizosaccharomyces pombe* and *Candida glabrata* possess the RIDD pathway, but lack any XBP1/HAC1-like transcription factors [90,91], and thus, their UPR-dependent transcriptional changes are solely regulated by RIDD in a single-component pathway [91,98]. In *Dictyostelium*, a considerable number of transcripts are downregulated in an IreA-dependent manner, thus suggesting the existence of a RIDD pathway. However, the ERS response also increases the abundance of specific transcripts, suggesting the existence of an IreA-dependent transcription factor; however, an XBP1 ortholog is yet unidentified [7]. Therefore, the *Dictyostelium* ERS response pathway may resemble that of the IRE1-branched animal mechanism.

### 3.2. IreA-Independent UPR Pathways in Dictyostelium

In contrast to the yeast UPR, in which the entire transcriptional response depends on the IRE1 pathway, in *Dictyostelium,* the response is only partially dependent on IreA [7], which suggests that, as in plants and animals, additional input signaling pathways must exist in this amoeba. The IreA-dependent transcriptional reprogramming primarily triggers an increase in the degradative capacity of the cell, and a decrease in protein load at the ER. As in yeast, the *Dictyostelium* IRE1 pathway regulates the expression of genes that participate in degradative processes, such as ubiquitin and ubiquitin ligases, together with ester-bond hydrolases and peptidases [7]. However, the expression of only a few ER chaperones depends on IreA, suggesting that, as in animal cells, IRE1-independent pathways may be implicated in the regulation of these components.

In animal cells, there are two UPR pathways that account for additional inputs, besides IRE1, to regulate the UPR (Figure 3). One of them is regulated by ATF6, a type II transmembrane ER-resident protein that bears a bZIP transcription factor domain in its cytosolic N-terminal portion. This UPR component is present in metazoan cells, and functional homologs were identified in plants (bZIP28 and bZIP17) [99,100]. Upon ERS, ATF6 is translocated from the ER to the Golgi body, where its transcription factor domain is released via a proteolytic cleavage triggered by the site-1 and site-2 proteases (S1P and S2P) [101,102]. The ATF6-dependent transcriptional reprogramming is required to induce the expression of genes that code for chaperones and degradative enzymes, and of genes involved in ER-membrane remodeling [103]. ATF6 signaling participates in senescence-associated ER expansion [104], and variants of this gene underlie the pathogenesis of inherited retinal and cone photoreceptor disorders [105,106].

In addition, in animals, the UPR is regulated by PERK, a transmembrane kinase that regulates protein translation through phosphorylating the eukaryotic initiation factor 2α (eIF2α) [107]. In plants, the presence of PERK orthologs is unreported.

Identification of ATF6 and PERK orthologs in *Dictyostelium* was unsuccessful; nevertheless, the existence of homolog components of these pathways cannot be ruled out. ATF6 belongs to the bZIP transcription factor family, and *Dictyostelium* contains a large number of these transcription factors. It was suggested that bZIPs evolved from a single common ancestor, which expanded and diversified greatly during evolution [108]. The current opisthokont bZIP transcription factors emerged from three ancestral groups that appeared during the evolution of this phylogenetic branch. One of these ancestral groups gave rise to the ATF6/HAC1 set of transcription factors, which is absent in other phylogenetic branches, such as the Amoebozoa or Plantae [108]. However, ER transmembrane bZIP transcription factors, which are activated upon ERS by a mechanism analogous to the one that regulates ATF6 and XBP1, were identified in plants [99,109]. *Dictyostelium* transcription factors may have diverged largely, so further characterization of the potential 19 bZIP transcription factors coded for in its genome may unravel the presence of ATF6 and XBP1 functional homologs.

## 4. ER Stress and the Autophagy Pathway in *Dictyostelium*

Degradative pathways also participate in homeostasis recovery upon ERS. Protein degradation is mainly achieved through two mechanisms. One is the ER-associated degradation (ERAD), which is accomplished by the proteasome, and thus, requires protein retro-translocation from the ER to the cytoplasm [110,111]. The other is autophagy, which delivers cytoplasmic material to the lysosome through double-membrane vesicles, known as autophagosomes [112]. If cells are not able to recover from ERS, the UPR represses the adaptive response, and triggers cell death [113].

In mammalian and plant cells, IRE1 activity is not only devoted to mRNA processing. In addition to the activation of its RNAse domain, IRE1 kinase can regulate other signaling pathways that orchestrate a more complex response. In animal cells, upon persistent ER stress, IRE1 can activate the Jun N-terminal kinase (JNK) by interacting with the adaptor protein TNF-receptor associated factor 2 (TRAF2) [114]. Depending on the severity of the stress, this signaling leads either to autophagy induction or to apoptosis activation [97,115]. Similarly, in plants, it was observed that the IRE1 kinase domain is required for the induction of autophagy upon ERS [116]. However, the JNK pathway is absent in *Dictyostelium*, and in other organisms outside the animal kingdom, such as plants or fungi [117,118]. The JNKs belong to the high osmolarity glycerol (HOG1)-like mitogen-activated protein kinase (MAPK) family, a group that emerged through duplication from a common ancestor that gave rise in fungi to a single HOG1 kinase. In contrast to p38, the other animal MAPK of this group, JNK genes underwent rapid evolution [118]. Thus, the IRE1-mediated JNK signaling, triggered by ERS in animal cells, might be a specialized trait of this phylogenetic group that emerged later during animal evolution.

Recently, we determined that autophagy is required for cell survival in response to ER stress in *Dictyostelium* cells [7]. However, in contrast with the animal and plant scenario, we found that IreA is not required for this autophagy induction. This suggests the presence of IRE1-independent pathways that may sense ERS and induce autophagy as a survival response. However, the IreA-mediated recovery of ER homeostasis was required to achieve a fully functional autophagy-dependent degradation, thus highlighting the functional connection of the ER with autophagosome biogenesis [7].

*Dictytostelium* cells not only lack JNK signaling pathways, but also caspase-dependent apoptotic cell death. However, there is a pathway of programmed cell death that is displayed by these amoebas with the characteristics of autophagic cell death (ACD) [119]. ACD is a death process characterized by cytoplasm vacuolization without chromatin condensation as in apoptosis, or organelle swelling as in necrosis [120]. It was described that ACD participates in cell death during *Drosophila* development [121], in hypersensitive cell death in plants [122], and in mammalian cell death under certain conditions [123]. Still, the nature and specific mechanisms that regulate ACD remain poorly defined.

In *Dictyostelium*, stalk cells die through ACD during fruiting-body formation [124,125]. Interestingly, research using this amoeba uncovered a novel link between the ER and ACD regulation, since it was observed that this type of cell death depends on the ER Ca^2+^-channel inositol 1,4,5-trisphosphate receptor (IP3R). Presumably, it is required to increase the cytosolic concentration of Ca^2+^ [126]. Since changes in calcium homeostasis at the ER might imbalance several cellular functions (for example, ER protein-folding and chaperone functions), studies on the participation of the UPR pathways in ACD regulation in this amoeba might unravel novel links between both pathways. It can also be inferred that, due to the absence of caspase-dependent apoptosis in *Dictyostelium,* the UPR may only be devoted to survival responses; thus, the study of the cellular effects of sustained ER stress in *Dictyostelium* may shed light on conserved survival responses that are hindered in animal cells by apoptotic signaling.

## 5. Assessing ER Stress in *Dictyostelium*

The onset of a stress response is usually evaluated by analyzing changes in the expression of marker genes. In *Dictyostelium*, ERS induces an increase in the abundance of several transcripts [7], whose levels can be analyzed to determine whether or not a defined treatment or growth condition leads to ERS. In Table 2, we present a selected list of IreA-dependent and independent genes, whose expression showed significant changes upon a TN treatment, and that can be useful as ERS markers. In addition, the changes in the expression of proteins such as cell division cycle protein D (CdcD), a conserved ATPase that participates in protein retro-translocation from the ER [127], can be evaluated via western blotting. It can also be determined whether a certain stimulus activates an IreA-dependent response, by following IreA clustering behavior in a time-lapse confocal microscopy assay of IreA-GFP-expressing cells that were exposed to the stimulus under study (Figure 4B).

**Figure 5:**
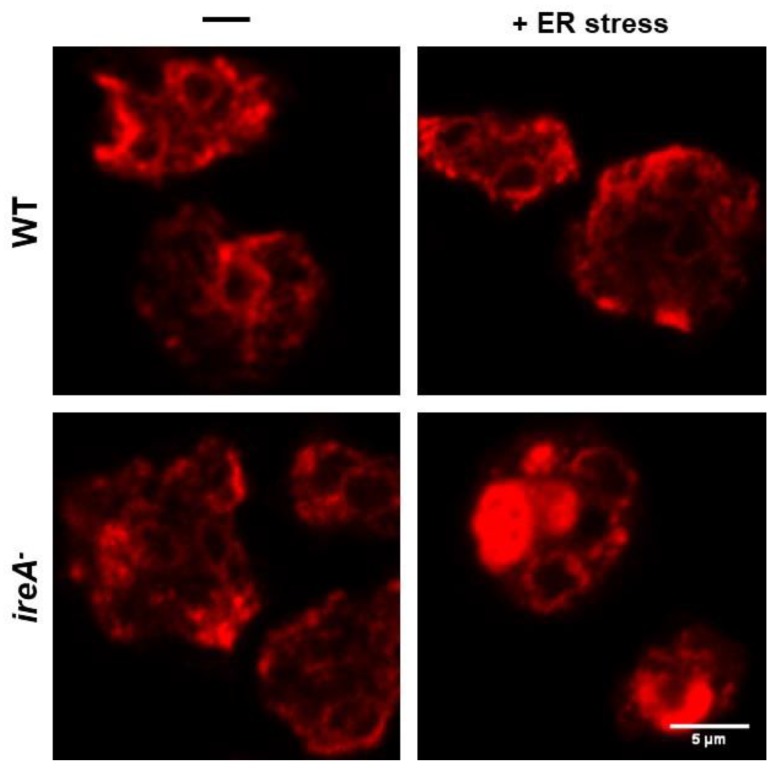
WT and *ireA^−^* cells, after an ER stress treatment or mock, were fixed and prepared for the detection of the ER-resident protein disulfide isomerase (PDI) via an immunofluorescence assay and were visualized using confocal microscopy. An ER stress treatment severely impaired the ER morphology of the sensitive *ireA^−^* cells. (Scale bar corresponds to 5 μm).

Changes in ER morphology upon ERS can be followed using immunofluorescence staining. Specific antibodies against *Dictyostelium* ER-resident proteins, such as calnexin and PDI [62,128], are available. As depicted in Figure 5, ER morphology defects in ERS-sensitive strains, such as the *ireA^−^* mutant, can be easily detected with this technique.

**Figure 6:**
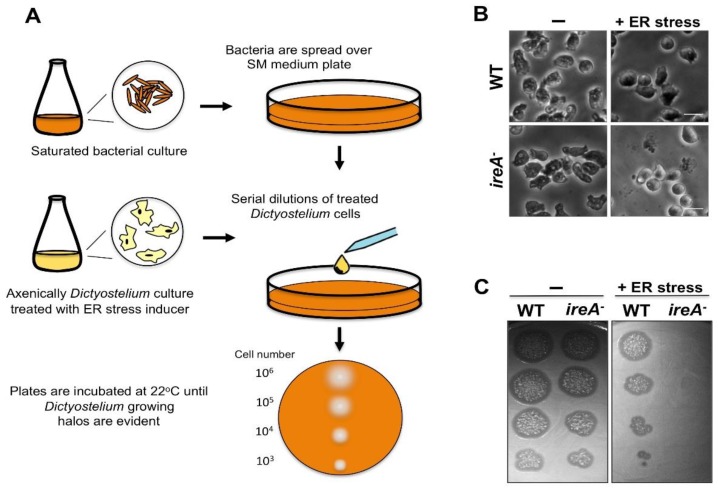
(**A**) Descriptive diagram of a serial dilution spotting assay used to test if a certain strain is sensitive to an ER stress inducer. A culture of bacterial cells (*Dictyostelium* is usually fed with *Klebsiella aerogenes* or *Escherichia coli*) is grown to saturation, and an aliquot is spread over an SM agar plate. Axenically growing *Dictyostelium* strains in the mid-logarithmic growth phase (with a density of around 1 × 10^6^ cells/mL) are prepared and treated for the desired times with the ER stress inducer. After the treatment, *Dictyostelium* cells are collected, and serial dilutions are prepared and spotted on the SM agar plates. Plates are incubated at 22 °C until lysis plaques emerge due to the presence of growing amoebas feeding on bacteria. (**B**) Light microscopy pictures of WT and ER stress-sensitive *ireA^−^* cells treated with a stress inducer. Morphological changes and cell lysis can be analyzed before the spotting assay. Notice the presence of round cells and the cell debris in the *ireA^−^* strain after the treatment. (**C**) Picture of a spotting assay where a WT and an ER stress-sensitive strain (*ireA^−^* cells) were tested with mock or ER stress inducer treatment.

The sensitivity of mutant strains to ER stressors, such as TN, can be evaluated via serial dilution spotting assays, as described previously [7] (Figure 6). This assay is performed by spotting serial dilutions of cells that were previously treated with various concentrations of the ERS inducer and/or for various treatment times, over agar plates with bacteria (Figure 6A). It is recommended to evaluate cell morphology before spotting cells (Figure 6B). After removal of the stressor, cells that survived the treatment can reinstate growth in association with bacteria. The inability of a certain strain to restore growth after treatment with ERS inducers reflects its sensitivity to this condition (Figure 6C). Currently, the only reported ER stress-sensitive strain is the *ireA^−^* mutant, which can be included as a control in this sort of assay [7].

## 6. Concluding Remarks

*Dictyostelium* proved to be an advantageous model in cell biology, and more recently, it emerged as a valuable organism for the study of ER-associated processes, such as the pathways involved in the ER stress response. These pathways show intriguing similarities, but also some differences between *Dictyostelium* and other organisms, expanding our knowledge of this conserved pathway across evolution. In addition, *Dictyostelium* poses an interesting model to unravel the role of proteins with unknown functions that are conserved in animals, but absent in yeast, and whose study may lead to a deeper understanding of how the complex regulation of the ER network is attained.

## Figures and Tables

**Table 1:** List of the endoplasmic reticulum (ER) protein orthologs mentioned throughout this text.

Function/Features	*Dictyostelium ^a^*	*Human ^a^*	*Saccharomyces cerevisiae ^a^*	*Arabidopsis thaliana ^a^*
***ER structure***				
Transmembrane protein that promotes membrane curvature, and participates in maintenance of tubular ER morphology	Reticulon-like group C (Rtnlc)/ Q54CA6	Reticulons-1 to 4 (RTN1 to RTN4)/ Q16799, O75298, O95197, Q9JK11	Reticulon-like proteins 1 (RTN1) and 2 (RTN2)/ A0A250W951, Q12443	Reticulon-like proteins B1 to 18, and 21 to 23 (RTNLB1 to 18 and RTNLB21 to 23)/ Q9SUR3, Q9SUT9, Q9SH59, Q9FFS0, O82352, Q6DBN4, Q9M145, Q9SS37, Q9LJQ5, Q6NPD8, Q9LT71, Q9M392, O64837, A2RVT6, Q9ZU43, Q8GYH6, Q6DR04, Q8LDS3, Q56X72, Q8GWH5, P0C941
Dynamin-like GTPase that mediates homotypic ER fusion	Sey1/ Q54W90	Atlastin-1 (ATL1)/ Q8WXF7	Sey1/ Q99287	Root hair defective 3 (RHD3) and root hair defective 3 homolog 2 (RHD3-2)/ P93042, Q9FKE9
***ER contact sites*** Components of the mitochondria encounter sites (ERMES), which are involved in the tether between the ER and the mitochondria to promote inter-organellar calcium and phospholipid exchange	Maintenance of mitochondrial morphology-1 (Mmm1)/ Q54MI5	ND	Maintenance of mitochondrial morphology protein 1 (Mmm1)/ P41800	ND
	Mitochondrial distribution and morphology-10 (Mdm10)/ Q54XQ5	ND	Mitochondrial distribution and morphology 10 (Mdm10)/ P18409	ND
	Mitochondrial distribution and morphology 34 (Mdm34)/ Q869R5	ND	Mitochondrial distribution and morphology protein 34 (Mdm34)/ P53083	ND
Transmembrane protein required to regulate ER contact sites, essential for autophagy and proper ER homeostasis	Vacuole membrane protein 1 (Vmp1)/ Q54NL4	Vacuole membrane protein 1 (VMP1)/ Q96GC9	ND	Vacuole membrane proteins 1 (KMS1) and 2 (KMS2)/ Q5XF36, F4I8Q7
***Lipid metabolism***				
Protein that associates to the lipid droplet surface	Perilipin (PlnA)/ Q54WC4	Perilipin proteins 1 to 5 (PLIN1 to 5)/ O60240, Q99541, O60664, Q96Q06, Q00G26	ND	ND
Catalyze the conversion of acyl coenzyme A (CoA) and 1,2-diacylglycerol to CoA and triacylglycerol.	Diacylglycerol *O*-acyltransferases 1 (Dgat1) and 2 (Dgat2)/ Q55BH9, Q54GC1	Diacylglycerol *O*-acyltransferases 1 (DGAT1), 2-acylglycerol *O*-acyltransferase 1 (MOGAT1)/ O75907, Q96PD6	Sterol *O*-acyltransferases 1 (Are1) and 2 (Are2)/ P25628, P53629	Diacylglycerol *O*-acyltransferase 1 (DGAT1)/ Q9SLD2
***Protein folding and modification***				
Subunits of the oligosaccharyl transferase complex, which catalyzes asparagine-linked glycosylation of newly synthesized proteins in the ER lumen	Oligosaccharyl transferase-1 (Ost1)/ Q54C27	Dolichyl-diphosphooligosaccharide-protein glycosyltransferase subunit 1 (RPN1)/ P04843	Dolichyl-diphosphooligosaccharide-protein glycosyltransferase subunit 1 (Ost1)/ P41543	Dolichyl-diphosphooligosaccharide-protein glycosyltransferase subunits 1A (OST1A) and 1B (OST1B)/ Q9SFX3, Q9ZUA0
	Oligosaccharyl transferase-2 (Ost2)/ Q54FB6	Dolichyl-diphosphooligosaccharide-protein glycosyltransferase subunit (DAD1)/ P61803	Dolichyl-diphosphooligosaccharide-protein glycosyltransferase subunit (OST2)/ P46964	Dolichyl-diphosphooligosaccharide-protein glycosyltransferase subunits 1 (DAD1) and 2 (DAD2)/ Q39080, O22622
	Oligosaccharyl transferase-3 (Ost3)/ Q54N33	ND	Dolichyl-diphosphooligosaccharide-protein glycosyltransferase subunit 3 (Ost3)/ P48439	Dolichyl-diphosphooligosaccharide-protein glycosyltransferase subunits 3A (OST3A) and 3B (OST3B)/ F4I8X8, Q9SYB5
	Oligosaccharyl transferase-4 (Ost4)/ Q54V54	Dolichyl-diphosphooligosaccharide-protein glycosyltransferase subunit 4 (OST4)/ P0C6T2	Dolichyl-diphosphooligosaccharide-protein glycosyltransferase subunit 4 (Ost4)/ Q99380	ND
	Oligosaccharyl transferase complex subunit C (Ostc)/ Q54X66	Oligosaccharyltransferase complex subunit OSTC (OSTC)/ Q9NRP0	ND	Oligosaccharyl transferase complex/magnesium transporter family protein (At4g29870)/ Q9SZQ8
	Wheat germ agglutinin-binding protein (Wbp1)/ Q54E62	Dolichyl-diphosphooligosaccharide-protein glycosyltransferase 48 kDa subunit (DDOST)/ P39656	Dolichyl-diphosphooligosaccharide-protein glycosyltransferase subunit (Wbp1)/ P33767	Dolichyl-diphosphooligosaccharide-protein glycosyltransferase 48 kDa subunit (OST48)/ Q944K2
	Suppressor of a *WBP1* mutation (Swp1)/ Q54HG9	Dolichyl-diphosphooligosaccharide-protein glycosyltransferase subunit 2 (RPN2)/ P04844	Dolichyl-diphosphooligosaccharide-protein glycosyltransferase subunit (Swp1)/ Q02795	Dolichyl-diphosphooligosaccharide-protein glycosyltransferase subunit 2 (RPN2)/ Q93Z16
	Staurosporine and temperature sensitivity (Stt3)/ Q54NM9	Dolichyl-diphosphooligosaccharide-protein glycosyltransferase subunits A (STT3A) and B (STT3B)/ P46977, Q8TCJ2	Dolichyl-diphosphooligosaccharide-protein glycosyltransferase subunit (Stt3)/ P39007	Dolichyl-diphosphooligosaccharide-protein glycosyltransferase subunits A (STT3A) and B (STT3B)/ Q93ZY3, Q9FX21
Heat shock protein 70 (Hsp70)-family chaperone	78 kDa Glucose-regulated protein (Grp78)/ Q8T869	Binding immunoglobulin protein/78 kDa glucose-regulated protein (BiP/Grp78)/ P11021	Binding immunoglobulin protein (BiP/Kar2)/ P16474	Binding immunoglobulin protein 2 (BIP2)/ F4K007
Hsp90-family chaperone	94 kDa Glucose-regulated protein (Dd-grp94)/ Q9NKX1	Endoplasmin (GRP94)/ P14625	ATP-dependent molecular chaperone (Hsp82)/ P02829	Endoplasmin homolog (HSP90-7)/ Q9STX5
Calcium-binding proteins with chaperone activity	Calreticulin (CrtA)/ Q23858	Calreticulin (CALR)/ P27797	ND	Calreticulin-1 (CRT1) and 2 (CRT2)/ O04151, Q388587
	Calnexin (CnxA)/ Q55BA8	Calnexin (CANX)/ P27824	Calnexin homolog (Cne1)/ P27825	Calnexin homolog 1 (CNX1) and 2 (CNX2)/ P29402, Q38798
ER luminal protein that catalyzes the formation and remodeling of protein disulfide bonds	Protein disulfide isomerases 1 (Pdi1) and 2 (Pdi2)/ Q86IA3, Q54EN4	Protein disulfide isomerases (P4HB), A4 (PDIA4), A3 (PDIA3), and A6 (PDIA6)/ P07237, P13667, P30101, Q15084	Protein disulfide isomerase (Pdi1)/ P17967	Protein disulfide isomerase-like proteins 1-1 (PDIL1-1), 1-2 (PDIL1-2), 2-2 (PDIL2-2), and 2-3 (PDIL2-3)/ Q9XI01, Q9SRG3, O22263, O48773
***Unfolded Protein Response***				
ER transmembrane serine and threonine kinase with ribonuclease activity that senses ER stress	Inositol-requiring enzyme A (IreA)/ Q55GJ2	Inositol-requiring enzyme proteins 1α (IRE1 α or ERN1) and 1β (IRE1β or ERN2)/ O75460, Q76MJ5	Inositol-requiring enzyme 1 (Ire1)/ P32361	Inositol-requiring enzyme proteins 1a (IRE1a) and 1b (IRE1b)/ Q93VJ2,
***Calcium channel*** Ion channel participates in calcium release from the ER, and is activated by inositol trisphosphate	Inositol 1,4,5-trisphosphate receptor (IplA)/ Q9NA13	Inositol 1,4,5-trisphosphate receptors type 1 (ITPR1), type 2 (ITPR2), and type 3 (ITPR3)/ Q14643, Q14571, Q14573	ND	ND

*^a^* Protein orthologs/ UNIPROT identifiers. ND, no homology detected.

**Table 2:** List of selected genes that showed a significant transcript increase upon a 16 h tunicamycin treatment, and that are suggested for evaluation as ER stress markers (list extracted from Domínguez-Martín, E. et al., 2018 [7]).

Gene ID	Name	Description	IreA-Dependent
**DDB_G0276445**	Grp78	Heat shock protein Hsp70 family protein.	no
**DDB_G0274199**	DDB_G0274199	Putative metallophosphoesterase.	no
**DDB_G0278477**	sarB	ADP ribosylation factors/ Secretion-associated and Ras-related (ARF/SAR) superfamily protein. GTP-binding protein Sar1B involved in vesicular transport between the endoplasmic reticulum and the Golgi body.	no
**DDB_G0283867**	*cprC*	Cysteine proteinase 3.	no
**DDB_G0278371**	*spc1*	Ortholog of the conserved microsomal signal peptidase 12 kDa subunit; the signal peptidase complex is a membrane-bound endo-proteinase that removes signal peptides from nascent proteins as they are translocated into the lumen of the endoplasmic reticulum.	no
**DDB_G0281833**	DDB_G0281833	Ubiquitin-conjugating enzyme E2.	no
**DDB_G0283113**	*eriA*	RNA exonuclease.	no
**DDB_G0290227**	*npl4*	Ortholog of nuclear protein localization 4 (NPL4), which, together with ubiquitin fusion degradation protein 1 (Ufd1) and cell division cycle protein D (CdcD), is involved in recognition of polyubiquitinated proteins, and their presentation to the 26S proteasome for degradation.	no
**DDB_G0287685**	*cinC*	Elongation factor 2. Translocates the peptidyl-tRNA from the aminoacyl site to the peptidyl site on the ribosome during protein synthesis; induced by cycloheximide; knockdown has significantly reduced ability for protein synthesis.	yes
**DDB_G0269462**	DDB_G0269462	Large protein containing two ubiquitin domains.	yes
**DDB_G0291121**	*cinB*	Esterase/lipase/thioesterase domain-containing protein.	yes
**DDB_G0285131**	derl2	Derlin-2. component of endoplasmic reticulum-associated degradation (ERAD) for misfolded luminal proteins.	yes
**DDB_G0270272**	*uae1*	Ubiquitin activating enzyme E1.	yes
